# Bioaccumulation of selected metals in bivalves (Unionidae) and *Phragmites australis* inhabiting a municipal water reservoir

**DOI:** 10.1007/s10661-013-3610-8

**Published:** 2014-01-10

**Authors:** Piotr Rzymski, Przemysław Niedzielski, Piotr Klimaszyk, Barbara Poniedziałek

**Affiliations:** 1 Department of Biology and Environmental Protection, Poznan University of Medical Sciences, Rokietnicka 8, 60-806 Poznań, Poland; 2 Department of Water and Soil Analysis, Adam Mickiewicz University, Umultowska 89B, 61-614 Poznań, Poland; 3 Department of Water Protection, Adam Mickiewicz University, Umultowska 89, 61-614 Poznań, Poland

**Keywords:** Heavy metals, Bivalve, Common reed, Bioaccumulation, Water reservoir

## Abstract

Urbanization can considerably affect water reservoirs by, inter alia, input, and accumulation of contaminants including metals. Located in the course of River Cybina, Maltański Reservoir (Western Poland) is an artificial shallow water body built for recreation and sport purposes which undergoes restoration treatment (drainage) every 4 years. In the present study, we demonstrate an accumulation of nine metals (Cd, Co, Cr, Cu, Fe, Mn, Ni, Pb, Zn) in water, sediment, three bivalve species (*Anodonta anatina*, *Anodonta cygnea*, *Unio tumidus*), and macrophyte *Phragmites australis* collected before complete drainage in November 2012. The mean concentrations of metals in the sediment, bivalves, and *P. australis* (roots and leaves) decreased in the following order: Fe > Mn > Zn > Cu > Cr > Ni > Pb > Co > Cd. A considerably higher bioconcentration of metals was observed in samples collected from the western and southern sites which undergo a higher degree of human impact. Sediments were found to be a better indicator of metal contamination than water samples. Interspecific differences in levels of metal accumulation were found between investigated unionids. *U. tumidus* accumulated higher levels of Cr, positively correlated with ambient concentrations, predisposing this species as a potential bioindicator of this metal in aquatic environments. On the other hand, species of *Anodonta* genus demonstrated higher accumulation of Cu and Cd. Positive correlations were found between Pb content in the sediments and tissues of all three bivalve species. In *P. australis*, metals were largely retained in roots except for Cd and Pb for which higher concentrations were found in leaves suggesting additional absorption of these metals from aerial sources. *P. australis* and bivalve from the Maltański Reservoir may be a potential source of toxic metals for animals feeding upon them and contribute to further contamination in the food chain.

**Electronic supplementary material:**

The online version of this article (doi:10.1007/s10661-013-3610-8) contains supplementary material, which is available to authorized users.

## Introduction

Municipal water reservoirs can be used for various recreational and sporting purposes and play an important role in urban landscape architecture. Situated close to the city centers and residential areas, they are usually under strong impact of their catchment areas and can accumulate a variety of pollutants including heavy metals. Although these elements are natural constituents of the earth’s crust, indiscriminate human activities have drastically altered their geochemical cycles and biochemical balance. In aquatic environments, heavy metals are produced from various natural and anthropogenic sources, such as atmospheric deposition, geologic weathering, agricultural activities, as well as residential and industrial products (Demirak et al. 2006). The contamination of aquatic ecosystems with heavy metals has become a serious worldwide problem. They are resistant to degradation under natural conditions and may accumulate in microorganisms and aquatic flora and fauna which, in turn, may enter terrestrial food chains (including human) and result in further contamination of the environment (Arnason and Fletcher 2003; Järup 2003; Milošković et al. 2013).

A broadly defined group of “heavy metals” is constituted of elements which are essential for living organisms in small quantities but toxic in higher concentrations (e.g., Cu, Fe, Mn, and Zn) and those which are not considered to have any specific metabolic role and are generally classified as toxic (e.g., Cd, Hg, and Pb) to living organisms (Singh et al. 2011). A wide range of adverse effects can be induced by heavy metals in biota and include alterations of growth, metabolic processes, and disease development (Järup 2003). Therefore, it is important to monitor their levels in the surface water of any human use. Recently, many studies have focused on the evaluation of trace metal bioaccumulation in the aquatic biota including microorganisms (Rzymski et al. 2013; Twining and Baines 2013), macroalgae (Rybak et al. 2012a, b, 2013), higher plants (Mishra et al. 2008; Obolewski et al. 2011), macroinvertebrates (Liu et al. 2010; Tunca et al. 2013), fish (Mason et al. 2000), and birds (Alhashemi et al. 2011; Wurtsbaugh et al. 2011).

The Maltański Reservoir (Poznań, Poland) is a small and shallow artificial water body, which is one of the flagships of the city and is considered to be one of the most beautiful regatta courses in Europe. However, its location near the city center, nearness of the heavy traffic roads and residential areas, as well as the flow-through character of the lake has a strong impact on its water quality. This results in high deposition of sediments which consequently shallows the reservoir thereby creating the necessity to remove sediments in order to sustain its sporting role. This procedure, supervised by the city council, is carried out every 4 years and is preceded by complete drainage of the reservoir, harvesting of fish, and partial removal of emerged plants dominated by *Phragmites australis*. As this kind of restoration was planned for November 2012, we undertook to investigate the bioaccumulation of trace metals in the biota of Maltański Reservoir. Our study presents the accumulation level of one of the most common and serious environmental metal pollutants (Cd, Co, Cr, Cu, Fe, Mn, Ni, Pb, Zn) in abundant bivalve and macrophyte species that had been built up in this municipal water body during the short period of time (4 years) since the previous restoration. We used water and sediment samples as the reference points. We also aimed to investigate whether these organisms could be applied in bioindication of metal pollution in municipal water bodies.

## Material and methods

### Description of the study area

The Maltański Reservoir (known also as the Malta Lake) situated in mid-western Poland (52°24′N, 16°58′E), near the center of the City of Poznań was built in 1952 by damming the River Cybina. It is a shallow (mean depth of 3.1 m, maximum depth of 5.0 m) and a small (area 64 ha) water body with an approximate volume of 2.1 million m^3^ (Gramowska et al. 2010). In the summer season, it is intensively used for national and international canoeing and rowing competitions and serves as a bathing area for city residents.

**Fig. 1:**
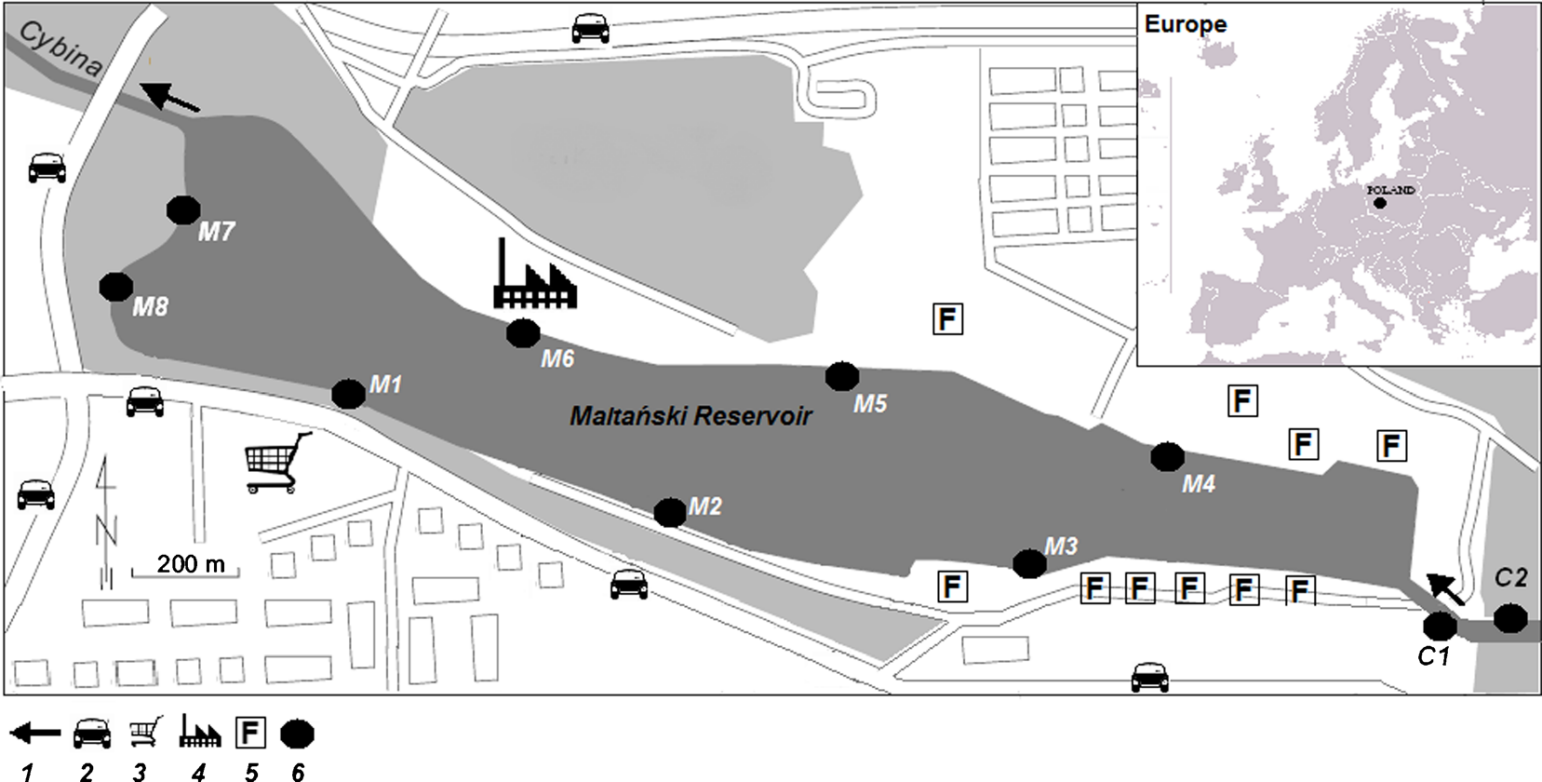
Location of the Maltański Reservoir and sampling sites. *1* water flow direction; *2* road with high traffic intensity; *3* shopping center; *4* distillery; *5* sport and recreation facility; *6* sampling site

Its whole water column is well mixed by westerly winds, which prevail in this region (Joniak et al. 2000). In the eastern part of the reservoir, the River Cybina flows into the lake and supplies it with nutrient-rich water with a prevalence of nitrates and phosphates that stimulates the primary production of phytoplankton in the Maltański Reservoir (Kozak and Gołdyn 2004; Gołdyn and Szeląg-Wasielewska 2005). Due to the morphometric features of the basin and westerly winds, the main water flow is directed near the northern shore. The eastern part of the reservoir is surrounded by forests, the southern by various sport, recreation, and shopping facilities as well as residential areas. The northern part is mainly surrounded by woods, an alcohol distillery, and sport facilities. Roads with high traffic intensity are situated to the western and southern parts of the reservoir (Fig. 1).

Catchment area of the Cybina River is dominated by cultivated fields. Before the river flows into the Maltański Reservoir, first it flows through the Lake Swarzędzkie (surface 93.7 ha) and four small man-made reservoirs—Antoninek, Młyński, Browarny, and Olszak (Kowalczewska-Madura 2003; Gramowska et al. 2010). Their surfaces are 7.2, 9.2, 7.1, and 3.3 ha, respectively; the mean water residence times are 0.5, 1.7, 1.4, and 0.6 days, respectively; and their maximum depths are 0.8, 2.3, 2.2, and 1.8 m, respectively (Gramowska et al. 2010). Until 1991, municipal and industrial sewage were discharged directly into Lake Swarzędzkie. After the implementation of urban sewage system, many buildings still have had illegal connections with a nearby ditch or with the rain drainage system while storm water was not collected nor treated until 2002 (Kowalczewska-Madura 2003). The lake remains degraded and hypertrophic with anoxia observed at the bottom during the summer season (Kowalczewska-Madura and Gołdyn 2006). Main sources of potential metals contamination in the Maltański Reservoir include combustion processes and transport as well as the Lake Swarzędzkie, which is heavily polluted with metals as already indicated by Gramowska et al. (2010)

### Sampling

Samples of water, bottom sediments, bivalves, and *Phragmites australis* (Cav.) Trin. ex Steud. 1841 (the common reed) were collected in parallel from eight sites (Fig. 1). Due to diversification of human pressure within the direct catchment area of Maltański Reservoir (as described in 2.1. subchapter), three sites were situated in the southern part of the reservoir (M1–M3), three in the northern part (M4–M6), and two in the western part (M7–M8). Samples were not collected from the eastern part of the reservoir due to a concrete bottom, a lack of *Phragmites australis* and technical difficulties (finish area of regatta course). Additionally, water and sediment samples (bivalve and *Phragmites australis* were not present) were collected from two sites at the River Cybina (C1–C2) located 100 and 200 m before it flows into the Maltański Reservoir. The samples were collected at the beginning of November 2012 just before the complete drainage of the reservoir.

The water was sampled near the surface (20-cm below the water table) into 0.5-L plastic bottles (Thermo Scientific, USA) and transported at 4 °C. In the laboratory, the samples were immediately filtered through GF/C filters (Whatman, UK), acidified with HNO_3 (_Sigma-Aldrich, Germany) for preservation, and deposited in 50-mL tubes at −20 °C. Three replicates from each sampling site were collected.

Sediments were collected using a plastic tube sediment sampler (diameter of 5 cm and length of 1 m). Only the surface layer of the sediment, approximately 5-cm thick, was collected for analysis. Three replicates from each sampling site were collected. The sediment was placed in a plastic container and transported to the laboratory in cold storage. The sediment was then sieved through a nylon sieve (mesh diameter of 500 μm) to remove plant debris, sand, stones, and other impurities (e.g., plastic and glass material). The resulting sediment fraction was dried for 2 h at 105 °C and placed in 125-mL plastic containers (Thermo Scientific, USA) for further mineralization.

Bivalve specimens were collected manually from 0.5 to 1.0-m depth, placed in a plastic bucket flushed with water from given sites and transported to the laboratory within 2 h. The following species were identified: *Anodonta anatina* (Linnaeus, 1758), *Anodonta cygnea* (L., 1758), and *Unio tumidus* (L., 1758). Five animals of each species representing the largest found individuals were collected from each sampling site. Mean shell length was 7.23 ± 0.87 cm for *A. anatina*, 7.51 ± 0.92 cm for *A. cygnea*, and 6.42 ± 1.02 cm for *U. tumidus*. Mussels were kept in the laboratory in continuously aerated deionized water (Milli-Q, Millipore, USA; resistivity 18.2 MΩ·cm) without food for several days to allow them to defecate. To prevent contamination before the shells were shucked, the external surface was thoroughly cleaned with brush and water to remove all sand and periphyton adhering to the shell. All bivalves were dissected using a plastic knife and the soft tissues were stored immediately at −20 °C. After thawing at room temperature and rinsing six times with deionized water, the soft tissues were dried to a constant weight for 24 h at 80 °C. Dried samples were weighed, homogenized by grinding in a porcelain mortar, and stored in plastic containers flushed with HNO_3_ (14 mol L^−1^) for further mineralization (Liu et al. 2010).

*Phragmites australis* samples were collected manually from the littoral area. From each site at the Maltański Reservoir, 5–6 samples of *Phragmites australis* were collected within a 5 × 2-m plot and placed in a plastic bag. In the laboratory, roots and leaves were washed thoroughly in deionized water to remove sediments and periphyton (Milošković et al. 2013). Plant material was then divided into two groups: (1) roots and (2) leaves, and dried to a constant weight at 80 °C for further mineralization (Obolewski et al. 2011).

### Sample mineralization

Samples of sediments, bivalve soft tissues, and *Phragmites australis* were subject to mineralization prior the analysis of metal concentration. 2.0 ± 0.1 g of sediment from each sample was placed in a conical flask and carefully flushed with 20 ml of HNO_3_ (14 mol L^−1^). Connected to a reflux condenser the flask was constantly heated at 80 °C for 30 min. After cooling the flask, its content was filtered using a cellulose filter (grade 1, 11 μm; Whatman, UK) into a polypropylene tube for analysis of metal concentration. A similar mineralization procedure was conducted for samples of *Phragmites australis* roots and leaves. Bivalves samples were kept for 14 days in containers flushed with HNO_3_ (14 mol L^−1^) then their contents were transferred to conical flasks and further prepared for metal concentration analysis as described for sediments and *Phragmites australis* samples. Procedure of sample digestion was adapted from EPA 3050B method (Environmental Protection Agency, USA).

### Analysis of metal concentration

The concentrations of Cd, Co, Cr, Cu, Fe, Mn, Ni, Pb, and Zn in the investigated samples were determined by the fast sequential atomic absorption spectrometer SpectrAA 220 FS (Varian, Australia) equipped with HCL lamps (Varian, Australia) and the Sampling System with Electronic Control Module SIPS-20 (Varian, Australia). The description of the applied conditions is given in Table A1. The calibration was performed using standard analytical solutions (Merck, Germany). Prior to the analysis, the detection method was validated with reference materials: LKSD-1 (sediment), ERM-CE278K (mussel), and BCR-060 (aquatic plant). The recovery rate exceeded 90 % for all determined elements, at low RSD values (below 5 %). The limit of metal detection was 0.01 mg L^−1^ for water samples and 0.01 mg kg^−1^ for sediments, bivalve and *Phragmites australis* samples.

### Calculations and statistical analysis

To evaluate the efficiency of metal bioaccumulation in bivalves and *Phragmites australis*, a biosediment accumulation factor (BSAF; Zhao et al. 2012) was calculated according to the equation:

$$ \mathrm{BSAF}={C}_x/{C}_s $$

where *C*_*x*_ represents the average concentration of metal determined in the organism and *C*_*s*_ represents the concentration determined in the associated sediment

To compare the total content of metals at the different sampling sites, the metal pollution index (MPI) was calculated according to the following equation (Usero et al., 1997):

$$ \mathrm{MPI}={\left(C{f}_1\times C{f}_2\dots C{f}_n\right)}^{1/n} $$

where *Cf*_*n*_ is the concentration of metal *n* in the sample.

Translocation of metals within the plant were evaluated by the translocation factor (TF; Mazej and Germ 2009), expressed by the following ratio: [trace element]_leaf_/[trace element]_root_.

The results were analyzed with Statistica 10.0 software (StatSoft, USA). Gaussian distribution was tested with Shapiro-Wilk’s test, and because most of the data did not meet its assumption, non-parametric methods were employed. The correlations were defined using Spearman’s rank correlation coefficient (*rs*). In order to compare two independent variables the Mann Whitney *U* test was used. Multiple comparisons were performed using the Kruskal-Wallis one-way analysis of variance with the Dunn post hoc test. A *p* value of <0.05 was considered as statistically significant.

## Results

### Metal concentrations in water

Relatively low concentrations of metals were detected in water samples from the Maltański Reservoir (Table 1). At all sampling sites, concentrations of Cd, Cu, and Pb were below the detection limit. Overall metals concentration in water generally ranked in decreasing order: Fe > Mn > Zn > Cr > Co > Ni. Statistically significant positive correlations were found between concentrations of Fe and Mn (*rs* = 0.81, *p* < 0.05), Fe and Zn (*rs* = 0.67, *p* < 0.05), and Mn and Zn (*rs* = 0.73, *p* < 0.05).

**Table. 1:** Mean values and standard deviation (SD) of metal concentrations in water (*n* = 24), sediments (*n* = 24), *P. australis* (*n* = 46), and bivalve (*n* = 40 each) from the Maltański Reservoir

	Water [mg L^−1^]		Sediment [mg kg^−1^]		*Phragmites australis* roots [mg kg^−1^]		*Phragmites australis* leaves [mg kg^−1^]		*Anodonta anatina* [mg kg^−1^]		*Anodonta cygnea* [mg kg^−1^]		*Unio tumidus* [mg kg^−1^]	
	Mean	SD	Mean	SD	Mean	SD	Mean	SD	Mean	SD	Mean	SD	Mean	SD
Cd	b.d.l.	–	0.09	0.01	0.08	0.03	0.11	0.01	0.04	0.03	0.08	0.03	0.04	0.02
Co	0.01	0.01	0.31	0.17	0.65	0.28	0.20	0.20	0.18	0.09	0.10	0.04	0.18	0.07
Cr	0.16	0.09	7.81	2.21	9.73	5.02	4.50	0.9	0.33	0.17	0.64	0.31	1.11	0.71
Cu	b.d.l.	–	11.36	3.92	11.86	4.89	5.23	1.37	9.33	1.12	5.25	2.26	4.59	2.43
Fe	2.54	1.55	4457.86	852.32	2081.00	1203.85	325.2	176.67	78.3	15.07	76.99	14.62	66.76	23.13
Mn	1.52	2.21	693.43	255.58	961.14	551.71	580.60	429.05	29.35	8.02	33.36	9.34	19.09	6.02
Ni	0.01	0.01	4.23	0.53	5.70	1.69	2.54	0.84	0.06	0.04	0.86	0.24	0.77	0.09
Pb	b.d.l.	–	2.73	1.3	1.63	1.07	1.67	0.44	0.15	0.07	0.16	0.05	0.21	0.11
Zn	0.64	0.31	71.96	18.98	71.84	36.25	62.32	20.49	42.21	11.39	31.11	11.6	51.29	25.22

*b.d.l*. below detection limit

Spatial difference in metal concentrations in water was observed for Fe, Mn, and Zn (Table 2). The highest content of Fe and Zn was detected in water sampled in the southern part of the reservoir. The western part revealed the highest concentration of Mn in water. The lowest average concentrations of Fe, Mn, and Zn were observed at the northern site of the reservoir.

**Table 2:** Mean values of metals concentrations in water (mg L^−1^), sediments, bivalve soft tissues, and roots and leaves of *Phragmites australis* (mg kg^−1^) in the relation to the collection sites and metal pollution index (MPI)

Sample	Area	Cd	Co	Cr	Cu	Fe	Mn	Ni	Pb	Zn	MPI
Water	S	b.d.l.	0.02	0.12	b.d.l.	**4.48NW**	**1.22NW**	0.02	b.d.l.	**0.99NW**	–
	N	b.d.l.	0.02	0.14	b.d.l.	**0.38SW**	**0.05SW**	0.01	b.d.l.	**0.41S**	
	W	b.d.l.	b.d.l.	0.26	b.d.l.	**0.70SN**	**7.40SN**	0.01	b.d.l.	**0.52S**	
Sediment	S	0.08	0.31	7.41	**10.08W**	**4560.00W**	**678.67W**	3.94	**3.36N**	**72.72N**	12.1
	N	0.09	0.30	8.07	**9.80W**	**4163.33W**	**641.67W**	4.24	**1.95SW**	**65.28W**	11.3
	W	0.11	0.36	8.26	**19.90SN**	**5020.00SN**	**893.00SN**	5.08	**3.19N**	**89.70N**	15.2
*Phragmites australis* roots	S	0.07	**0.79NW**	**7.92NW**	**9.99W**	**2044.00NW**	**921.25NW**	**5.07W**	**2.50N**	**81.10NW**	12.7
	N	b.d.l.	**0.29SW**	**3.93SW**	**7.74W**	**251.00SW**	**453.00SW**	**3.91W**	**1.17SW**	**54.70SW**	5.1
	W	0.09	**0.53SN**	**16.25SN**	**17.65SN**	**3070.00SN**	**1295.00SN**	**7.85SN**	**2.13N**	**101.50SN**	16.5
*Phragmites australis* leaves	S	0.11	**0.59NW**	**4.70NW**	**6.14N**	**224.00W**	**773.00N**	**2.59N**	**2.01N**	**47.10NW**	7.5
	N	b.d.l.	**0.14S**	**3.66W**	**3.66SW**	**260.50W**	**203.50SW**	**1.93N**	**1.13SW**	**59.40SW**	3.7
	W	0.10	**0.07S**	**5.79N**	**5.79N**	**440.50SN**	**861.50N**	**3.12SN**	**2.08N**	**79.00SN**	7.1
*Anodonta anatina*	S	0.024	**0.20**	**0.45N**	**10.11W**	**83.68W**	**35.30W**	**0.17NW**	**0.17N**	**35.90W**	1.6
	N	0.049	**0.10**	**0.19SW**	**8.56W**	**72.24W**	**23.39W**	**0.04S**	**0.04SN**	**35.40W**	1.0
	W	0.06	**0.14**	**0.40N**	**11.50SN**	**106.00SN**	**52.10SN**	**0.12S**	**0.13N**	**50.29SN**	1.8
*Anodonta cygnea*	S	0.12	**0.10**	**0.89NW**	**5.89W**	**44.34W**	**28.10W**	**1.13NW**	**0.19**	**29.25W**	2.0
	N	0.04	**0.15**	**0.29SW**	**3.60W**	**46.90W**	**16.21W**	**0.70S**	**0.11**	**22.70W**	1.3
	W	0.07	**0.06**	**0.74SN**	**7.91SN**	**172.00SN**	**61.01SN**	**0.74S**	**0.21**	**43.20SN**	2.3
*Unio tumidus*	S	0.04	**0.20**	**1.37NW**	**5.25W**	**62.01W**	**18.65NW**	**1.32W**	**0.21**	**41.41W**	2.1
	N	b.d.l.	**0.15**	**0.68SW**	**3.28W**	**54.37W**	**9.73SW**	**b.d.l.**	**b.d.l.**	**36.50W**	0.7
	W	0.02	**0.21**	**0.83SN**	**7.74SN**	**109.00SN**	**32.40SN**	**0.20S**	**0.20**	**61.16SN**	1.8

Bolding indicates the significance difference in metal concentrations between sites (Kruskal-Wallis H test, *p* < 0.05). Letters specify which sites differ from each other (Dunns post hoc test, *p* < 0.05)

Sampling sites: *S* southern site (M1–M3); *N* northern site (M4–M6); *E* western part (M7–M8), *b.d.l*. below detection limit

Water samples collected from the River Cybina also revealed low metal concentrations (Table A2). At both sites (C1–C2), Cd, Co, Cr, Cu, and Pb content were below the detection limit. Fe, Mn, and Zn were the dominant metals.

### Metal concentrations in sediments

All studied metals were detected in the sediment samples collected from the Maltański Reservoir at all sampling sites (Table 1). The overall mean concentrations of metals decreased in the following order: Fe > Mn > Zn > Cu > Cr > Ni > Pb > Co > Cd. Concentration of Mn was positively correlated with Fe (*rs* = 0.80, *p* < 0.05) and Zn (*rs* = 0.81, *p* < 0.05), Cd with Cr (*rs* = 0.79, *p* < 0.05), whereas Pb with Cu (*rs* = 0.55, *p* < 0.05). Concentration of Ni was positively correlated with Cr (*rs* = 0.63, *p* < 0.05), Cu (*rs* = 0.54, *p* < 0.05), Fe (*rs* = 0.67, *p* < 0.05), Cd (*rs* = 0.64, *p* < 0.05), and Zn (*rs* = 0.57, *p* < 0.05).

Several spatial differences in metal concentrations in the sediment from the Maltański Reservoir were observed (Table 2). The highest concentration of Cu, Fe, Mn, and Zn was noted in the western part of the reservoir. When compared with northern and southern sampling sites, the mean concentration of Cu was higher by 10.1 and 9.8 mg kg^−1^, Fe by 856.7 and 455.0 mg kg^−1^, Mn by 214.3 and 251.3 mg kg^−1^, and Zn by 24.4 and 17.0 mg kg^−1^, respectively. The northern part of the reservoir accumulated the lowest concentrations of Cu, Mn, Fe, Pb, and Zn in the bottom sediments. No significant differences in spatial distribution of metals in sediments were noted for Cd, Co, Cr, and Ni (Table 2).

All the studied metals were also detected in sediments collected from the River Cybina (Table A2). Their content generally exceeded values found in the Maltański Reservoir. Statistically significant differences in metal concentrations in sediments were found for Co, Cr, Fe, Mn, Ni, Pb, and Zn (*p* < 0.05). When compared to the Maltański Reservoir, the mean content of metals in River Cybina sediments was higher by 0.46 mg kg^−1^ for Co, 5.20 mg kg^−1^ for Cr, 772.79 mg kg^−1^ for Fe, 142.77 mg kg^−1^ for Mn, 0.97 mg kg^−1^ for Ni, 5.97 mg kg^−1^ for Pb, and 21.58 mg kg^−1^ for Zn.

### Metal concentrations in bivalve soft tissues

All the studied metals were detected in the soft tissues of three bivalve species collected from the Maltański Reservoir (Table 1). The overall mean concentrations of metals decreased in the following order: Fe > Mn > Zn > Cu > Cr > Ni > Pb > Co > Cd. The mean detected accumulation level in bivalve species amounted to 0.05 mg kg^−1^ (±0.02) for Cd, 0.15 mg kg^−1^ (±0.05) for Co, 0.69 mg kg^−1^ (±0.39) for Cr, 6.39 mg kg^−1^ (±2.37) for Cu, 74.02 mg kg^−1^ (±6.32) for Fe, 27.27 mg kg^−1^ (±7.36) for Mn, 0.56 mg kg^−1^ (±0.44) for Ni, 0.17 mg kg^−1^ (±0.03) for Pb, and 41.54 mg kg^−1^ (±10.11) for Zn. Several statistically significant correlations between metal concentrations were found. Mn was positively correlated with Cu (*rs* = 0.59, *p* < 0.05), Fe (*rs* = 0.61, *p* < 0.05), and Zn (*rs* = 0.60, *p* < 0.05). On the other hand, a negative correlation was found between Cr concentration and Cu (*rs* = −0.59, *p* < 0.05) and Mn (rs = −0.52, *p* < 0.05).

Several differences in the spatial distribution of metals in bivalve soft tissues were observed for each studied species (Table 2). Cu, Mn, and Fe revealed the highest content in samples of *A. anatina*, *A. cygnea*, and *U. tumidus* collected from the western part of the reservoir. For *A. cygnea* and *U. tumidus*, the content of Fe in individuals from the western site was twice as high as that from the northern and southern sites. The highest levels of Cr were noted in *A. anatina*, *A. cygnea*, and *U. tumidus* inhabiting the southern part of the reservoir. The bioaccumulation of Ni was also considerably higher in all the studied bivalve species from the southern part. It was particularly obvious for *U. tumidus* whose soft tissues averagely contained over sixfold higher concentrations of this metal in the southern part than in the western (Ni content was below the detection limit in *U. tumidus* from the northern part of the reservoir). Generally, as demonstrated by MPI, the lowest concentrations of metals in soft tissues of the studied bivalve species were observed in the northern part of the reservoir. Ni, Cd, and Pb were below the detection limit in *U. tumidus* collected at this site (Table 2).

Interspecific differences in levels of metal accumulation were also found. The highest concentrations of Cu were observed in *A. anatina* and amounted on average to 9.33 mg kg^−1^, higher by 4.08 mg kg^−1^ than in *A. cygnea* and 4.75 mg kg^−1^ than in *U. tumidus*. These differences were statistically significant (*p* < 0.05). On the other hand, soft tissues of *U. tumidus* contained higher levels of Cr than in those of *A. anatina* and *A. cygnea* (*p* < 0.05). The mean differences in Cr level amounted to 0.78 mg kg^−1^ when compared to *A. anatina* and 0.47 mg kg^−1^ when compared to *A. cygnea*. The mean content of Zn in the soft tissues of *U. tumidus* was also higher than in *A. anatina* (by 9.08 mg kg^−1^) and *A. cygnea* (by 20.18 mg kg^−1^) (Table 1) but no statistical significance was found (*p* > 0.05).

**Fig. 2:**
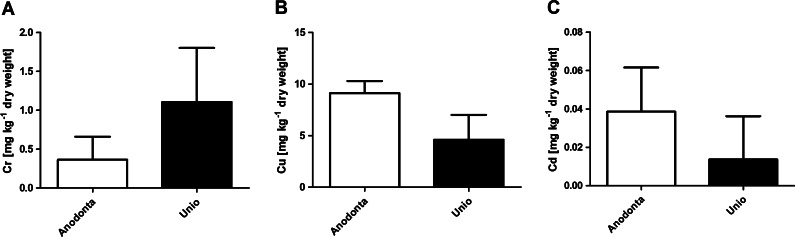
Comparison of Cr (**a**), Cu (**b**), and Cd (**c**) content (mean ± SD) in bivalve species from *Anodonta* (*n* = 80) and *Unio* (*n* = 40) genus collected from the Maltański Reservoir. All differences are statistically significant (Mann Whitney *U* test, *p* < 0.05)

We also compared metal concentrations between two genera of the investigated bivalve species. *Unio* accumulated considerably higher levels of Cr (Fig. 2a), whereas soft tissues of *Anodonta* contained higher levels of Cu (Fig. 2b) and Cd (Fig. 2c). All differences were statistically relevant (*p* < 0.05).

The accumulation of metals in the soft tissues of the investigated bivalve species were lower than in sediment samples, thus the calculated BSAF values did not exceed unity in any case. The highest values of BSAF (>0.7) were observed for Cu in *A. anatina*, Cd in *A. cygnea*, and Zn in *U. tumidus*. Cr, Fe, Mn, and Pb demonstrated extremely low BSAF values (<0.15) in all the investigated species (Table 3).

**Table 3:** Mean values of Biosediment Accumulation Factor (BSAF) for *Phragmites australis* and bivalve species and translocation factor (TF) in *P. australis* from the Maltański Reservoir

	*Anodonta anatina*	*Anodonta cygnea*	*Unio tumidus*	*Phragmites australis*		
	BSAF			BSAF roots	BSAF leaves	TF leaves/roots
Cd	0.41	0.81	0.44	0.81	1.16	1.43
Co	0.58	0.57	0.33	2.08	0.65	0.31
Cr	0.04	0.08	0.14	1.25	0.58	0.46
Cu	0.82	0.46	0.40	1.04	0.46	0.44
Fe	0.02	0.02	0.01	0.47	0.07	0.16
Mn	0.04	0.05	0.03	1.39	0.84	0.61
Ni	0.02	0.20	0.18	1.35	0.60	0.44
Pb	0.06	0.06	0.08	0.60	0.61	1.02
Zn	0.59	0.43	0.71	1.01	0.87	0.87

The correlation coefficients between metal content in tissues of different bivalve species and ambient concentrations are listed in Table 4. Concentration of Pb in sediments was positively correlated with its content in the tissues of all three investigated species. We also found positive correlations between Cr content in ambient samples (water and sediment) and its content in *U. tumidus* tissues.

**Table 4:** The Spearman rank correlation coefficients between metal concentrations in ambient samples and soft tissues of bivalve species and *Phragmites australis*

	Water					Sediment				
	*A. anatina*	*A. cygnea*	*U. tumidus*	*P. australis* roots	*P. australis* leaves	*A. anatina*	*A. cygnea*	*U. tumidus*	*P. australis* roots	*P. australis* leaves
Cd	–	–	–	–	–	0.22NS	−0.20NS	−0.31NS	0.37*	0.22NS
Co	−0.06NS	−0.15NS	−0.41NS	0.16NS	−0.28NS	0.31NS	0.01NS	−0.08NS	0.07NS	0.23NS
Cr	0.1NS	−0.15NS	0.74**	0.51*	0.36NS	0.05NS	0.09NS	0.59*	0.59*	−0.34NS
Cu	–	–	–	–	–	−0.32NS	0.23NS	0.12NS	0.89**	0.11NS
Fe	0.06NS	−0.02NS	−0.05NS	0.18NS	0.40NS	0.25NS	0.01NS	0.08NS	−0.18NS	−0.28*
Mn	0.38NS	0.17	0.72*	−0.13NS	0.10NS	0.21NS	−0.24NS	0.15NS	−0.19NS	0.63**
Ni	−0.06NS	0.57	−0.40NS	0.44*	−0.28*	0.60*	−0.34NS	0.09NS	0.96**	0.11NS
Pb	–	–	–	–	–	0.57*	0.57*	0.59*	0.35*	0.20NS
Zn	−0.36NS	−0.01	0.27NS	0.10NS	−0.27NS	−0.04NS	−0.29NS	0.20NS	−0.10NS	−0.26NS

Bolding indicates statistically significant correlations

*NS* not significant (at 0.05 level)

*p < 0.05; **p < 0.01

### Metal concentrations in *Phragmites australis*

All studied metals were detected in both roots and leaves of *Phragmites australis*. Fe and Mn were dominant elements reaching >2,050 and >950 mg kg^−1^ in roots and >300 and >550 mg kg^−1^ in leaves, respectively (Table 1). The overall mean concentrations of metals in roots and leaves decreased in the following order: Fe > Mn > Zn > Cu > Cr > Ni > Pb > Co > Cd. Statistically significant positive correlations between concentrations of Mn and Cu (*rs* = 0.61, *p* < 0.05) and Mn and Zn (*rs* = 0.65, *p* < 0.05) were found. Concentration of Ni was positively correlated with Co (*rs* = 0.59, *p* < 0.05), Cr (*rs* = 0.87, *p* < 0.05), Cu (*rs* = 0.93, *p* < 0.05), and Fe (*rs* = 0.77, *p* < 0.05).

**Fig. 3:**
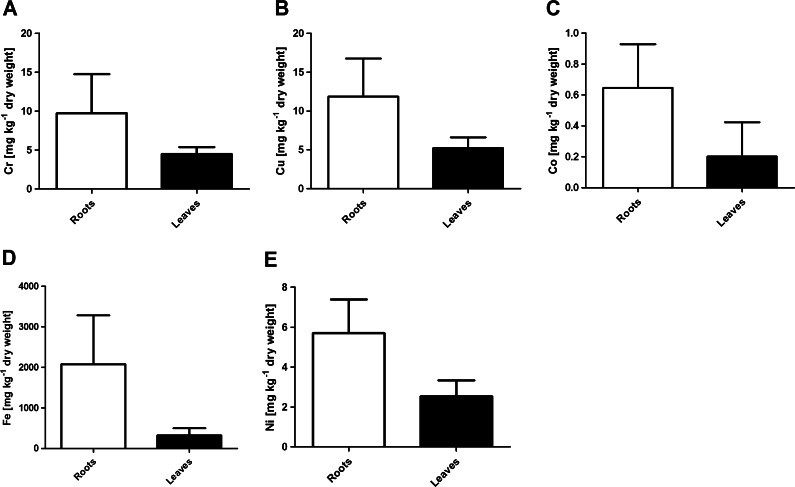
Comparison of Cr (**a**), Cu (**b**), Co (**c**), Fe (**d**), and Ni (**e**) content (mean ± SD) in roots and leaves of *Phragmites australis*. All differences are statistically significant (Mann Whitney *U* test, *p* < 0.05)

Statistically significant differences were also found in corresponding metal concentrations in roots and leaves. Roots accumulated significantly higher levels of Cr, Cu, Co, Fe, and Ni (Fig. 3a–e). However, the mean concentration of Cd and Pb in leaves was higher than in roots (Table 1), no statistical differences in metal content between these organs were found (*p* > 0.05).

There were several spatial differences in metal content determined in roots and leaves from the Maltański Reservoir (Table 2). The highest contents of Cr, Cu, Fe, Mn, Ni, and Zn were found in roots collected from the western part of the reservoir with Fe content being over tenfold higher than in roots from the northern sites. In leaves, similar spatial differences were observed for Cr, Fe, Mn, Ni, and Zn. On the other hand, the highest levels of Co were found in the roots and leaves collected from the southern site. Levels of Pb in both roots and leaves of *Phragmites australis* collected in the southern and western parts of the reservoir were comparable but significantly higher than those determined in samples from the northern part. Generally, as demonstrated by MPI, the lowest metal concentrations were found in the roots and leaves of *Phragmites australis* collected from the northern part with Cd level below the detection limit (Table 2).

Calculated TF indicates that elements accumulated by *Phragmites australis* were largely retained in roots as shown by a general value of factor <1. High mobility was shown only for Cd and Pb and resulted in TF values of >1 (Table 3). Comparison of BSAF values calculated separately for roots and leaves confirmed that most of the metals were accumulated in roots. BSAF >1 values in *Phragmites australis* roots were observed for Cr, Cu, Mn, Co, Ni, and Zn with the highest value found for Co (>2). In leaves, BSAF value >1 was only found for Cd (Table 3).

The correlation coefficients between metal content found in roots/leaves of *Phragmites australi*s and ambient concentrations are listed in Table 4. Strong positive correlations were found between Cu and Ni contents in roots and their respective sediment concentrations. Both Cd and Pb also revealed a statistically significant positive correlation between their sediment concentration and that found in roots.

## Discussion

The Maltański Reservoir has already been described as a sink of nutrients (Gołdyn et al. 2001) and therefore, we decided to investigate whether various metals (essential and toxic) are also largely deposited in this water body. As suggested by Gramowska et al. (2010), Lake Swarzędzkie and Cybina River can play a relevant role in the metal contamination of this reservoir although only the metal content in water was a subject to investigation (Gramowska et al. 2010). Considering that the bioaccumulation of trace metals is highly situation-dependent, a thorough estimation of its level requires some general information on metal concentration in the habitat of the studied biota and identification of any potential external sources of metals in the water body. Therefore, beside water, we also investigated metal content in sediments and collected samples from different sites varying in human impact.

Unsurprisingly, metal concentrations in sediments were higher than those in water samples in which some (Cd, Pb) were even below the detection levels. Because metal content in water can be a subject to seasonal variations and may not accurately reflect the actual problem of contamination, more reliable data on the availability of these elements can be provided by sediment investigations (Tekin-Özan 2008; Duman and Kar 2012). Both types of organism employed in our study (bivalves and macrophytes) are not only largely abundant in the Maltański Reservoir but their habitat remains highly related to sediments. The unionids are mobile, burrowing suspension-feeders while *Phragmites australis* is a perennial emergent macrophyte that takes up elements mainly from sediments through its roots. It is noteworthy that apart from metal concentration in sediments, their bioavailability and uptake is a complex function of many factors including water pH, redox potential, temperature, hardness, nutrients concentration, total organic content (both particulate and dissolved fractions) (Boudou and Ribeyre 1997). As demonstrated by Rybak et al. (2013), Cu and Zn accumulation in *Ulva* from the Maltański Reservoir was positively correlated with total dissolved solids content, but negatively with water temperature. On the other hand, increase in conductivity and nitrates resulted in simultaneous decrease in Cu and Mn accumulation, respectively. Contradictory correlations were, however, found for the river *Ulva* while other authors demonstrated differences in metal uptake between various aquatic animals and plants species inhabiting ecosystems of similar physical and chemical parameters (Usero et al. 1997; Duman et al. 2007; Rybak et al. 2013). Therefore both, the aqueous chemistry and the physiology of the living organisms can be important in affecting metal bioavailability.

At metal concentrations that are within ranges common to freshwater, some bivalve species have already been suggested as an effective bioaccumulators (Elder and Collins 1991). The important advantages of these organisms in such applications are their large size, limited mobility, abundance in freshwater environments, and relative ease of collection and species identification (Szefer 2002). Marine species have been successfully adopted to monitor elemental contaminants (e.g. trace metals, radionuclide) in many coastal areas worldwide (Szefer 2002; Lehtonen et al. 2006; Benedicto et al. 2011). Recently, several studies have also focused on the reflection of ambient contamination using freshwater representatives of the Unionidae family in lakes and rivers (Gundacker 2000; Liu et al. 2010; Pourang et al. 2010).

In our study, the degree of metal accumulation in soft tissues followed the same order and spatial variations as in the associated sediments thereby indicating a strong relationship between ambient and biological concentrations. This is in general agreement with observations from other investigations on metal bioaccumulation involving unionids. However, levels of metals found in the soft tissues of *A. anatina*, *A. cygnea*, and *U. tumidus* were relatively low in our study when compared to those found in *Anodonta woodiana* (Królak and Zdanowski 2001; Liu et al. 2010) or *Unio pictorum* (Gundacker 2000). According to Szefer et al. (1999), bioaccumulation is expected to occur in organisms if the BSAF value is >1. In our study, BSAF value was below unity for every investigated metal indicating no obvious bioaccumulation of these elements in the studied species. On the other hand, our study represents a unique situation in which determined concentrations of metals are the result of contamination, deposition, and bioaccumulation occurring over the 4 years since the last drainage and sediment removal in 2008. Interestingly, we have reported an over tenfold higher mean concentration of Cu (in tissues of all three investigated species) than Pourang et al. (2010) in *A. cygnea*. However, the same authors also reported that Cu content was in negative correlation with specimen age. Cu as well as Fe, Mn, and Zn, are essential elements for bivalves although their demand and bioconcentration appears to be controlled by biological processes (Bordin et al. 1992; Moura et al. 2000). Cu forms hemocyanin, Fe binds to ferritin, an important detoxification protein, Mn is a cofactor for enzymes including manganese superoxide dismutase and Zn is required with more than 200 metalloenzymes for maximum catalytic activity (Bootsma et al. 1988; Eisler 1993, van Holde et al. 2001; Wang et al. 2013). Kraak et al. (1993) demonstrated that *U. pictorum* was able to regulate its internal Cu content within limited ranges of ambient concentrations while Moura et al. (2000) suggested that *A. cygnea* can efficiently detoxify excessive concentrations of Cu. Thus, this may indicate that the specimens in our study were still at an intensive development stage and had high metabolic demand on essential elements that resulted in a high capacity to accumulate Fe, Mn, Zn, and Cu in their tissues. This is also supported by statistically significant positive correlations that were found in our study between Mn and Cu and Fe and Zn content. Other studied metals—Cd, Co, Cr, Ni, and Pb, can be generally classified as toxic to bivalves and other organisms. Their bioconcentration depends mostly on environmental levels and not biological demands (Bordin et al., 1992). All unionid species investigated in our study colonize similar habitats; therefore, one could anticipate similar bioaccumulation of these elements. However, we have demonstrated that *U. tumidus* had a higher capacity to accumulate Cr and furthermore, that Cr content in its tissues was positively correlated with ambient concentrations (both water and sediment). This suggests that *U. tumidus* could be considered as a potential bioindicator of Cr contamination in aquatic environments. On the other hand, species of *Anodonta* genus demonstrated a higher susceptibility to Cd contamination despite its low ambient concentrations. It may, therefore, be suggested that species of the *Anodonta* genus rather than *Unio* may be good bioindicators of early Cd contamination in aquatic reservoirs. Despite low concentrations of Pb, we found significant positive correlations between metal content in sediments and tissues of all three bivalve species. Our finding is supported by the Gundacker (2000) study in which Pb content in the viscera, mantle, and adductor of *Anodonta* sp. was positively correlated with Pb concentrations in surface layers of sediments. Interestingly, in that study, no correlation between Pb in sediments and organs of *U. pictorum* was found indicating interspecific differences in the accumulation of this metal among representatives of the Unio genus. Our findings suggest that both studied *Anodonta* species and *U. tumidus* may be potential bioindicators of Pb contamination in aquatic environments.

Compared to bivalve tissues, higher levels of metal were found in *Phragmites australis*. This macrophyte is among those characterized by high resistance to environmental conditions, so it is common worldwide (Du Laing et al. 2006). In our study, *Phragmites australis* demonstrated a high capacity to bioaccumulate Co, Cr, Cu, Mn, Ni, Fe, and Zn in roots, reflected in BSAF values above unity. Among these, Cu, Mn, and Fe are essential metals intervening in several metabolic processes, mainly in photosynthesis, thus their content in leaves may be lowered at the end of the growing season (Duman et al. 2007; Quan et al. 2007). A particularly high value of BSAF (>2) was observed for Co, a metal present in water and sediment samples in very low quantities. It is noteworthy that all metal levels (essential and non-essential) found in roots and leaves were below reported phytotoxic levels (Marschner 1995; Kabata-Pendias and Pendias 1992; Pais and Jones 2000). With the exception of Cd and Pb, higher metal content was found in roots suggesting that these are primary metal accumulation organs. Furthermore, mobility of the studied elements was low indicating that roots act like filters for certain metals and protect aboveground tissues from contamination and toxicity. This observation has also been confirmed by other studies that have investigated bioaccumulation of metals in *Phragmites australis* and other macrophytes including *Typha angustifolia* L. subsp. *australis* (Schum. & Thonn.) Graebn., *Najas marina* L., *Potamogeton lucens* L., *Nuphar lutea* L., *Scirpus maritimus* L., or *Potamogeton nodosus* Poir., in which the uptake trend of trace elements decreased as root > stem > leaf (Mazej and Germ 2009; Alhashemi et al. 2011). Numerous correlations found in our study between metal content in roots and their respective concentrations in sediments suggest that underground rather than aboveground parts of *Phragmites australis* are better bioindicators of aquatic contamination. However, our study demonstrates that Cd and Pb content in leaves can exceed that found in roots. Although Cd is not a prerequisite for plant growth, it has been demonstrated that it is passively and metabolically taken up while its transport is regulated by vascular tissues (Prasad 1995). On the other hand, Pb translocation is low probably due to some physiological barriers against metal transport to aerial tissues (Kabata-Pendias and Pendias 1992) and this usually results in higher Pb deposition in the underground parts of *Phragmites australis* (Duman et al. 2007; Alhashemi et al. 2011). Contrary to this hypothesis, Fitzgerald et al. (2003) reported that *Phragmites australis* growing in Ireland accumulated significantly higher levels of Pb in shoots than roots. Kozłowska et al. (2009) found that accumulation of Cd and Pb in leaves of *Phragmites australis* occurred at a higher level in a pond situated within the urban area. Furthermore, it is worth highlighting that Pb can also be absorbed to some extent by leaves from aerial sources (Sharma and Dubey 2005). We did not investigate air quality in our study although it was noticeable that the highest Pb content was observed in leaves collected from sites surrounded by a high degree of human activity (western and southern). Moreover, a relatively high content of Cr and Ni was found in leaves. Their accumulation in this organ can result in further contamination of animals (e.g., insects) feeding upon the aboveground parts of *Phragmites australis*.

Metal concentration in bivalves and *Phragmites australis* generally followed the level of contamination of their immediate environment, particularly, that found in sediment samples. Despite the small size, depth, and volume of the Maltański Reservoir, spatial differences in the level of metals were found. The greatest differences were found for Cr, Cu, Fe, Mn, and Zn concentrations in sediment, bivalves and *Phragmites australis*. As expected, the lowest metal pollution was found in the northern part of the Maltański Reservoir which is characterized by the lowest degree of human activity. Southern and western sampling sites located near traffic and residential areas demonstrated, in turn, higher levels of metal accumulation. This suggests that the city surroundings have a great impact on the water quality of the Maltański Reservoir. For instance, it has been shown that urban runoff may be a source of various chemical compounds including metals and can contribute to contamination of an aquatic environment and its biota including mussels (Gillis 2012; Zgheib et al. 2012). Furthermore, we have demonstrated that sediments collected from the River Cybina contained significantly higher levels of metals than those found in samples from the Maltański Reservoir. This would indicate that the River Cybina constitutes a notable source of metals for the investigated water body. The main flow of water in the Maltański Reservoir is directed near the northern sides of the reservoir while the southern side is characterized by low water flow. This, in turn, stimulates higher rates of metal deposition along the southern sides and further bioaccumulation of these elements. This finding is also supported by the studies of Rybak et al. (2012a, 2012b, 2013) who investigated metal accumulation in *Ulva* collected from the same reservoir and observed noticeably lower concentrations in samples collected from northern parts (when compared to southern). This was particularly evident for Cd, Co, Cr, Cu, Mn, Ni, and Cu and is consistent with most of the differences obtained in our study.

## Conclusions

Our study demonstrates that artificial water bodies situated within urban areas can accumulate metals, including non-essential and toxic. Despite the relatively modest size of the Maltański Reservoir, spatial differences in metal distribution in both sediments and biota were observed and were related to the degree of human activity within the surrounding areas. Bivalves have been revealed to be useful in biomonitoring metal contamination in aquatic ecosystems including those undergoing restoration treatments although species-specific susceptibility needs to be taken into account. In our study, *U. tumidus* demonstrated a promising capability to accumulate Cr that could find an application in indicating contamination with this metal. *A. anatina* and *A. cygnea* revealed a potential for Cd bioindication. All bivalve species were found to be good potential indicators of Pb despite its relatively low content in the environment. Moreover, *Phragmites australis*, the dominant macrophyte in the Maltański Reservoir, was demonstrated to be a bioaccumulator of metals (particularly Cd, Co, Cr, Cu, Mn, Pb, and Zn). Most of the studied metals were largely retained in roots. Only Cd and Pb were found in leaves at comparable or even higher levels suggesting potential absorption of these metals from aerial sources. Relatively high concentrations of Cr and Ni were also found in leaves. Therefore, *Phragmites australis* may be a potential source of toxic metals for animals feeding upon this plant and contribute to further contamination in the food chain.

## Electronic supplementary material

Below is the link to the electronic supplementary material.

ESM 1 (DOC 31 kb)

ESM 2 (DOCX 12 kb)
