# Supplementary material for: Bioaccumulation of selected metals in bivalves (Unionidae) and Phragmites australis inhabiting a municipal water reservoir
Source: Environ Monit Assess. 2014 Jan 10;186(5):3199–212. doi: 10.1007/s10661-013-3610-8 (PMC3969812; doi:10.1007/s10661-013-3610-8)
Supplement: Supplementary file 1 — (DOC 31 kb) [file 10661_2013_3610_MOESM1_ESM.doc]

Table A1. The conditions applied in atomic absorption spectrometry (AAS) analyses.

| **Metal** | **Wavelenght**  **[nm]** | **Slit**  **[nm]** | **Deuterium background correction** | **Electric current**  **[mA]** |
| --- | --- | --- | --- | --- |
| Cd | 228.8 | 0.5 | yes | 5 |
| Co | 240.7 | 0.2 | yes | 10 |
| Cr | 357.9 | 0.2 | yes | 10 |
| Cu | 324.8 | 0.5 | yes | 10 |
| Fe | 248.3 | 0.2 | yes | 10 |
| Mn | 279.5 | 0.2 | yes | 10 |
| Ni | 232.0 | 0.2 | yes | 10 |
| Pb | 217.0 | 1.0 | yes | 5 |
| Zn | 213.9 | 1.0 | yes | 5 |
