# Supplementary material for: Bioaccumulation of selected metals in bivalves (Unionidae) and Phragmites australis inhabiting a municipal water reservoir
Source: Environ Monit Assess. 2014 Jan 10;186(5):3199–212. doi: 10.1007/s10661-013-3610-8 (PMC3969812; doi:10.1007/s10661-013-3610-8)
Supplement: Supplementary file 2 — (DOCX 12 kb) [file 10661_2013_3610_MOESM2_ESM.docx]

Table. A2. Mean values and standard deviation (SD) of metal concentrations in water and sediments from the River Cybina collected 100 m and 200 m before its flow into the Maltański Reservoir.

|  | **Water**  **[mg L^-1^]** | | **Sediment**  **[mg kg^-1^]** | |
| --- | --- | --- | --- | --- |
|  | **mean** | **SD** | **mean** | **SD** |
| **Cd** | b.d.l. | - | 0.11 | 0.05 |
| **Co** | b.d.l. | - | 0.77 | 0.08 |
| **Cr** | b.d.l. | - | 13.01 | 1.43 |
| **Cu** | b.d.l. | - | 11.89 | 3.31 |
| **Fe** | 0.54 | 0.36 | 5230.65 | 432.32 |
| **Mn** | 0.32 | 0.23 | 836.20 | 137.43 |
| **Ni** | 0.03 | 0.01 | 5.2 | 0.63 |
| **Pb** | b.d.l. | - | 8.7 | 1.20 |
| **Zn** | 0.21 | 0.05 | 93.54 | 12.54 |

b.d.l. – below detection limit.
